# New design of a cementless glenoid component in unconstrained shoulder arthroplasty: a prospective medium-term analysis of 143 cases

**DOI:** 10.1007/s00590-012-1109-6

**Published:** 2012-10-27

**Authors:** D. Katz, J. Kany, P. Valenti, P. Sauzières, P. Gleyze, K. El Kholti

**Affiliations:** 1 Clinique du Ter, 56270 Ploemeur, France; 2 Clinique de l’Union, 34240 Saint Jean, France; 3 Clinique Jouvenet, 75016 Paris, France; 4 2 rue de la Concorde, 68000 Colmar, France; 5 Clinique du Tonkin, 69100 Villeurbanne, France

**Keywords:** Glenoid, Osteoarthritis, Shoulder, Total shoulder arthroplasty, Uncemented metal back

## Abstract

The uncemented glenoid implants in total anatomical shoulder arthroplasty are likely to be accused of problems like dissociations, secondary rotator cuff tear, and wear of polyethylene (PE). This work is a clinical and radiological prospective review of 143 cases of anatomical total shoulder arthroplasty using a new metal back uncemented glenoid implant (MB) in order to see if this new implant induces those complications. A total of 143 cases were operated between 2003 and 2011. In a first part, the whole series of 143 cases was radiologically studied in order to quantify the lateralisation induced by the MB implant. In a second study, 37 cases had a mean follow-up of 38 months (24–75, mean 32) and served for the clinical and radiological final study. Pre- and postoperative clinical evaluation was done using the Constant–Murley score and the simple shoulder test from Matsen. The final X-rays served to detect an eventual secondary narrowing of the joint space and to analyse the frequency of radio lucent lines (RLL) and loosenings. Despite a small radiological lateralisation in comparison with the normal contralateral side (0.36 cm, *p* = 0.02), the clinical results after 2 years were similar to the published cemented glenoid implants series but without any RLL, glenoid loosening or joint narrowing. Some dissociations occured in the beginning and definitely eliminated by a design modification of the PE tray. The discussion tried to show that, despite a still short follow-up, this series is encouraging to continue to use this new MB implant. Different applications of the concept of universality and conversion are discussed, this tray been also the support of a glenosphere in reverse arthroplasty.

## Introduction

The gold standard technique for glenoid replacement in total anatomical shoulder prosthesis is still the use of full polyethylene cemented implant like Neer concept 40 years ago [1].

The main problem is glenoid loosening. Despite good and predictable clinical results, the frequency of radio lucent lines (RLL) is high, 70 % in the most recent literature with an increasing number of glenoid loosenings, 40 % at 10 years [2–4]. However, the number of revisions is low, around 5 % [5, 6].

These worrisome findings have spurred development of new ideas. Among them cementless glenoid devices have been tried, as much as the success of reverse prosthesis has obliged to develop metal back screwed glenoid trays, able to resist to the shear forces induced by a glenosphere [7].

These new uncemented implants in anatomical shoulder replacement have been greatly criticised, accused to be responsible of loosening, dissociation, and early PE wear. Furthermore, it has been advocated that the increased thickness induced by the metal tray [8, 9] could be a risk for the rotator cuff [10].

The excellent primary fixation of these screwed implants in reverse arthroplasty has led us to extend our indications to anatomical replacements, in order to see whether with this kind of design we are able to decrease the frequency of RLL observed with the cemented glenoids.

## Materials and methods

The metal back glenoid implant (MB) of the Universal Shoulder Arthroplastic System ARROW (FH orthopedics, 3 rue de la Forêt 68990-Heimsbrunn-France) is 6.5 mm thick, 3.5 for the PE and 3 for the metal tray. The deep convexe surface and the keel are covered with hydroxyapatite.

Four sizes are available 44, 46, 48 and 50. Whatever the size of the humeral head, there is a systematic mismatch between the radius of curvature of the glenoid and of the humeral head with an average of 4 mm (between 1 and 6).

The ancillary system allows a precise preparation of the glenoid with a reaming of the bone surface and a press fit preparation of the keel grove, in order to insure a perfect contact between hydroxyapatite and bone.

The primary fixation is insured by 2 axial screws and can be enhanced by a third sagittal screw. This third screw goes through an anterior plate and the keel. It can be useful in case of osteoporotic patient and glenoid bone loss, allowing an easy bone graft fixation.

On the humeral side, noncemented press fit stems were preferentially used, with grafting of the metaphysis using some cancellous bone from the humeral head. In case of osteoporotic bone, a classical cemented technique was recommended.

The clinical analysis included a pre- and postoperative evaluation of the Score of Constant and Murley [11], of the active and passive range of motion and of the simple shoulder test from Matsen [12].

Radiographic preoperative assessment consisted of plain anteroposterior radiographs with medial, neutral and lateral rotation, axillary and outlet view under fluoroscopic guidance. A systematic CT scan completed the preoperative radiographic analysis to evaluate the status of the cuff and the glenoid bone stock according to Walch classification [13].

Postoperative radiological study included an AP view with a standardised fluoroscopic technique and the X-ray beam perpendicular to the plane of the joint space. This allowed to detect an eventual narrowing, witness of a progressive polyethylene wear.

The study included 2 parts:

1. A radiological study done on the first postoperative X-rays in order to check if the increased thickness of the MB component induced a measurable lateralisation. The lateral offset was measured between the centre of the glenoid bone and the lateral border of the great tuberosity (Fig. 1). This measurement was compared with the normal contralateral side, if not involved.

**Fig. 1:**
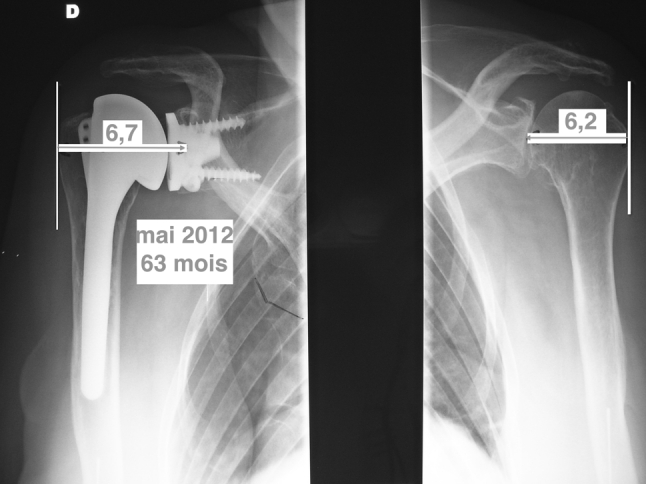
Technique of the measurement of the lateral offset
2. The secund part consisted of analysing the final clinical and radiological results on the patients with more than 24 months of follow-up.

The Student’s *t* test was used for statistical analysis when two groups had to be compared. When the comparison involved more than two groups, a variance analysis was applied. The chosen level of significance (*p*) was set at 0.05.

## Results

From November 2003 to December 2011, 143 total anatomical shoulder arthroplasties have been performed. Aetiology is summarised in Table 1, dominated by primary osteoarthritis with 90 % of normal cuff.

**Table 1:** Aetiology for the whole series

	Arthritis without tear	Arthritis with tear	Posttrauma arthritis	Revision	Chronic dislocation	Secondary necrosis	Malunion
MB (*n* = 143)	116 (81.1 %)	16 (11.2 %)	4	4	1	1	1

Delto pectoral approach was performed except in 2 cases.

The common size for the MB was 44 in 3/4 of the cases, and 46 for the remaining. The humeral stem size was mainly 10 or 12. 25 % of the humeral heads were 44, 25 % for 46 and 48. 65 % were excentric heads with a height of 16 for half of them.

Most of the biceps tendons were tenodesed (76 %).

Radiological analysis:

### 1. Radiological results

The results of the first part of this work are summarised in the Table 2. Seventy-eight patients had no bilateral involvement. The postoperative immediate radiological study showed a lateralisation of 0.36 cm between the operated and the contralateral normal side (*p* = 0.02).

**Table 2:** Study of the lateralisation, only for unilateral pathologic involvement with normal contralateral side and excluding the bad X**-**ray

	Lateralisation	Control side	Difference
MB (*n* = 48)	6.09 (3.1–7, mean 6.3)	5.73 (2.2–7, mean 6)	0.36 (*p* = 0.02)

With more than 2 years of follow-up 2 cases presented a well-tolerated superior migration. One, with a follow-up of 24 months, had no pain, an active flexion of 90° and a Constant score of 53 (68 %). The other, at 44 months, presented a slight pain, an active elevation of 100° and a score of 54 (74 %).

In the whole series of 143 MB prosthesis, we noted 4 cases of superior migration (2.8 %). Two were reoperated. All were anterior to 2008.

In 5 cases, the inferior screw was under the scapula with no clinical or radiological consequences.

Part of the cohort allowed a study of the joint narrowing. At 3 months, the difference between operated and contralateral normal side was 0.47 cm (*n* = 30, 0–1), at 1 year, we found a 0.44 of average (−1 to 1), at 2 years 0.42 (0–1) and finally 0.39 (−0.1 to 1) with no statistical significance. No lucent lines were recorded.

### 2. Clinical results

**Fig. 2:**
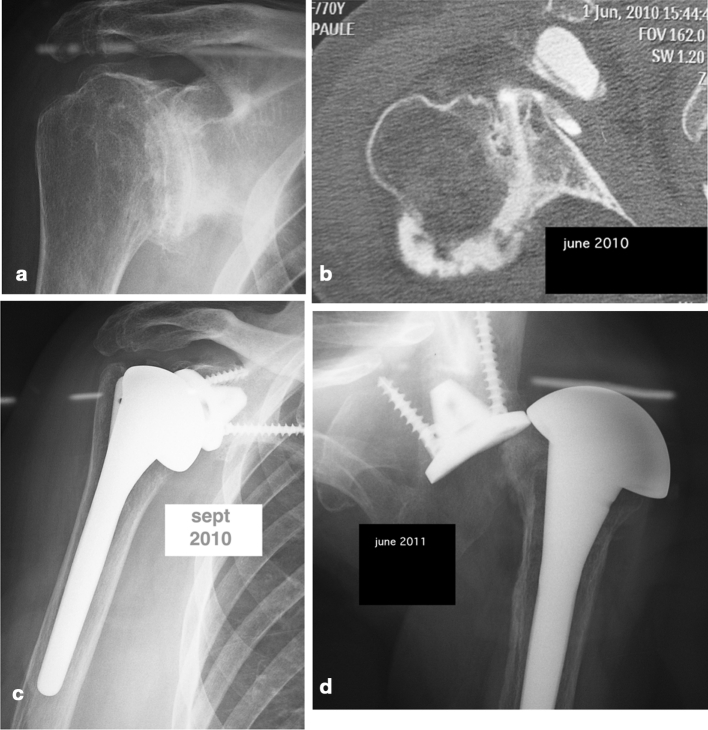
MB glenoid implanted on a type b2. A dislocation occured at 6 months. Conversion for a reverse shoulder arthroplasty: **a** preoperative X-ray, **b** preoperative CT scan showing the posterior subluxation, **c** immediate postop X-ray, **d** posterior dislocation at 6 months
- Two secondary rotator cuff tears were converted to a reverse. The first one, at 17 months of the revision, had no pain but a fair active and passive mobility with a Constant score of 44 (68 %). The other one was a b2 glenoid and had also a contralateral MB glenoid with an excellent result. During revision performed at 2 years a posterosuperior, PE wear was found with a contact between the metal tray and the humeral head. At 36 months of the revision, the result was excellent with a Constant score of 64 (100 %), an active elevation of 140° (Fig. 3).

Among the 143 cases, 11 complications were recorded (7.7 %):

- Three dissociations occured in the beginning of the experiment. A first design did not allow to precisely centre the PE tray before impaction. In 2007, the addition of a PE central peg allowed to insure a good alignment. This modification erased definitely this complication. Two of these cases were revised, one with a cemented glenoid with an excellent final Constant score of 85 (121 %) and an active flexion of 160°. The other one is 60 months of follow-up of a simple reimpaction of a new PE tray with also an excellent result, a Constant score of 90 (103 %) and an active elevation of 170°. The third case of dissociation did not accept any revision.
- Three dislocations occured, all with b2 and c glenoid types. Two were revised with a conversion in reverse. In one case with a type b2 glenoid, the conversion consisted of simply changing the PE tray to a glenosphere and the humeral head to a cup. Neither the MB tray nor the humeral stem was modified. This patient with 18 months of follow-up had an active flexion of 120°, no pain and a Constant score of 54 (76 %) (Fig. 2). For the 2 cases with a glenoid type c, because of the necessity of grafting the glenoid it was necessary to take out the metal tray despite a good integration. But like the type b2 case the humeral stem was untouched.

**Fig. 3:**
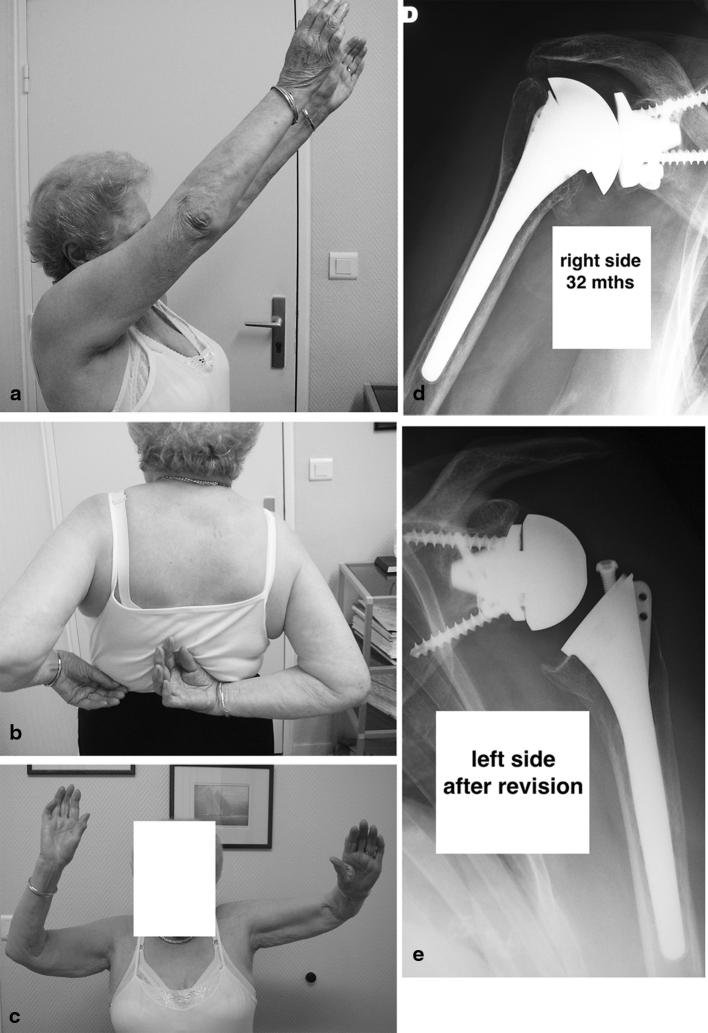
Patient operated on both sides with a metal back glenoid implant. No problem for the right side. On the left side a secondary cuff tear occured. A conversion for a reverse was realised with a good clinical result **a**–**c** clinical results at 36 months, **d** X-ray on the right side **e** X-ray on the left side after the revision
- One case sustained a superior migration due to a bad initial choice of the size of the glenoid with a too low implantation. This allowed a quick superior migration of the humeral head above the glenoid implant but without cuff tear. The revision at 1 month consisted on a higher implantation of a bigger metal tray. The MB 44 was converted to 48. This patient was 60 months of follow-up, and this case of revision was included in the final review.
- One patient had a postoperative painful stiffness because of a complex regional pain syndrome.
- Another case of painful shoulder was reoperated at 6 months. No aetiology was found. The MB glenoid was converted to a cemented glenoid. At 13 months, there was a superior migration with a bad result.

Globally, the revision rate was 8/143 = 5.59 %. But if we do not take account of the dissociation cases, the percentage decreases to 4.19 % (6/143).

### 3. Results after 24 months or more

Thirty-seven cases in 36 patients were 2 years of follow-up or more, 38.3 months in average (24–75, mean 32), 28 female (one bilateral) 8 men, 21 right side, and 32 right handed. The average age was 69 (35–83, mean 72). Aetiology was mainly primary osteoarthritis without cuff tear (Table 3). Four patients were already operated on, 2 subacromial decompression and 2 previous arthroplasties. The first one was this already mentioned patient with a too small MB glenoid implanted too low and revised at 3 months. The second one sustained previously a total anatomical arthroplasty with a cemented glenoid, revised with a MB glenoid and a grafting because of a glenoid loosening.

**Table 3:** Aetiologies for the cases with more than 24 months

	Arthritis without tear	Arthritis with tear	Posttrauma arthritis	Revision	Chronic dislocation
MB (*n* = 37)	32	1	1	2	1

### 4. Three revisions were recorded

- Two presented a superior migration due to a rotator cuff tear. One was already ruptured preoperatively, and the other was checked as pathologic. As already mentioned, these 2 cases were converted to a reverse, simple and rapid procedure thanks to the universality of the ARROW system, consisting of a simple change of the intermediate devices. These 2 cases were excluded from the final review.
- One patient presented an early dissociation revised at 2 months. A simple change of the PE tray allowed an excellent result at 39 months with an active flexion of 170° and a Constant score of 85 (102 %).
- One patient died from a medical cause in January 2012 after the final review in November 2011. Her result was excellent, 150° of flexion and a score of 72 (101 %).

Finally, 35 cases were available for the final results which are summarised in Table 4. Pain increased from 1.6 to 13.4, flexion from 92° to 146° and Constant score from 27 (36 %) to 70 (95 %). The statistical difference between pre- and postoperative values was greatly significant.

**Table 4:** Pre- and postoperative Constant score: the pre- and postoperative comparison is highly significant

MB (*n* = 35)	Pain	ADL	Strength	Constant score	Active elevation	RE1	RE2	SST
Preop	1.6 (0–5)	8.8 (2–18)	2.8 (0–10, med 2.5)	27 (12–56.36 %)	92° (40–160 mean 90)	12.5° (20–70)	27.6 (0–80, mean 30)	2.6 yes
Postop	13.4 (5–15, mean 15)	17.9 (9–20, mean 20)	7 (0–17, mean 6)	70 (35–90.95 %)	146° (80–180, mean 150)	44° (10–70 mean 45)	65 (10–95 mean 70)	9.8 yes

We analysed the results according to the preoperative type of glenoid:

- Between types A1 and A2 the preoperative clinical values were systematically inferior for the most used glenoids with no statistical significance. The same differences were noted postoperatively excepted for the activities of daily living.
- We found the same differences between A1 and B1 and between type A and type B.
- if we study the types A1 and B1 versus A2, B2 and C, the values were systematically lower pre- and postoperatively for the most used glenoids.

## Discussion

In total shoulder anatomical arthroplasty using a cemented glenoid, the percentage of radio lucent lines is high despite a good and stable clinical results [14] Whatever the model, keeled or pegged, at 10 years, 76 % presented a RLL and 40 % a glenoid loosening. The modular second or third generation tried to give a better adaptation to the patient’s anatomy but did not solve the problem [15, 16]. One of the most recent publication, with more than 5 years of follow-up, showed 18.9 % of loosenings, among them 23 % were progressive with a functional repercussion [17].

Many modifications of the glenoid devices tried to solve this problem. Parallely, the feasibility of the metal back trays, able to sustain without failure the shear forces induced by a glenosphere in reverse shoulder arthroplasty, led to experiment these devices in anatomical arthroplasty [18]. With this kind of design, the literature evoqued early polyethylene wear, number of dissociation between PE and metal tray, loosenings and superior migration due to rotator cuff tears. The first referred cause of those failures was the thickness of the implant [4, 19, 20]. However, most of the articles referred to old conceptions [21–23] or designs which did not insure a good primary fixation [24]. However, some more recent publications presented some encouraging results [25, 26].

In 1992, we started to work on a new implant which was available for clinical use in human since 2003. The convexity was preferred to a flat back tray, being widely recognised as insuring a good bone-implant contact and transforming the shear forces in compressive forces [1, 23, 27–30]. Iannotti [29], like Neer previously [1], insisted in the better easiness of well positioning the convexe implant, which parallely decreases the frequency of lucent lines [31]. The principle of a mismatch was also adopted [15, 22, 24, 25].

In our experience, the fit induced by a precise ancillary system and the frontal and the eventual saggital screwing avoided any primary fixation problems and enhanced the indications to the cases necessitating a glenoid bone graft. The hydroxyapatite coverage on all the parts in contact with the glenoid bone insured a good secondary fixation with no migrations, loosenings or even RLL.

We had on the beginning some cases of dissociations between PE and MB. A modification of the design, consisting of adding a small central peg on the PE allowed to well centre the PE before impaction on the metal tray. No more dissociation happened after this modification.

The total thickness of the implant is 6.5 mm. Our results showed an increasing in the lateral offset in comparison with a normal contralateral side. Another work (accepted as a free paper in the SOFCOT 2012 meeting) comparing the results between MB and cemented glenoid showed that this induced lateralisation did not influence the clinical results. Radiologically, no loosening, no lucent lines, no narrowing of the joint space, witness of a polyethylene wear were found. However, our tendency is actually to increase the PE thickness [26].

The rigidity of the metal back device was suspected to induce stress shielding and osteolysis under the metal [24, 32]. These experimental publications [33], in our knowledge, were never confirmed by clinical studies [34] and not confirmed in our work.

Some rotator cuff tears happened in our series, but with the same frequency than the most recent publications on cemented glenoid designs [17].

Despite the fact that, in the contrary than the literature [35, 36], the results were not statistically different between types a and b, the type of preoperative glenoid bone wear influenced our choice, as the only cases of dislocations were on posteriorly used glenoid bones, type b2 or c. In those cases where the posterior wear is not too important, we recommend to increase the anterior reaming, to add a posterior bone graft and eventually to utilise the long keeled implant, designed for revisions.

In old patients with a thin cuff, a cemented glenoid can be preferred to avoid any tension on the tendons. However, we prefer the noncemented glenoid, as, in case of secondary superior migration, the conversion in a reverse is facilitated, the same metal back tray been the support of the glenosphere. Moreover, this new design, thanks to the anterior plate and the 2 available directions of screwing, allows to extend the indications to big glenoid bone loss necessiting a bone graft [37].

However, this study presented some limitations:

- This is a medium-term study with a mean follow-up of 3 years.
- The measurement of the radiological lateral offset is technician dependent, and no scoring of the RLL has been used.
- This is a multicentric not randomised study, and the choice of either a noncemented or a cemented prosthesis was often subjective and surgeon dependent .

**Fig. 4:**
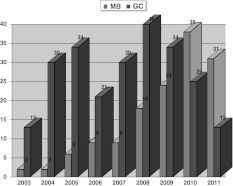
Diagram showing the growing number of Mb glenoid implantations

However, in the view of our good and predictable results, our indications of using a MB glenoid in total anatomical shoulder arthroplasty are increasing (Fig. 4).

In conclusion, we did not find in this medium-term clinical and radiological review of a noncemented metal back glenoid implant, the classical complications pointed out in the literature for those uncemented glenoid implants.

With this new design, despite a radiological increase in the lateral offset, there is no proved risk for the cuff, no early polyethylene wear, no dissociation and the clinical results seem to be similar to the cemented glenoids but avoiding their frequent troubles such as evolutive lucent lines and loosenings.
